# Molecular characterization of an analphoid supernumerary marker chromosome derived from 18q22.1➔qter in prenatal diagnosis: a case report

**DOI:** 10.1186/s13039-014-0069-4

**Published:** 2014-10-22

**Authors:** Vincenzo Altieri, Oronzo Capozzi, Maria Cristina Marzano, Oriana Catapano, Immacolata Di Biase, Mariano Rocchi, Giuliana De Tollis

**Affiliations:** Department of Genetics, ASL Napoli 1 Centro, Napoli, Italy; Department of Biology, University of Bari, Bari, Italy; Diagnostic Service Srl, Casoria, Napoli Italy; MeriGen Research Srl., Napoli, Italy

**Keywords:** Small supernumerary marker chromosomes, Neocentromere, Prenatal diagnosis, FISH analysis

## Abstract

**Background:**

Small supernumerary marker chromosomes (sSMC) occur in 0.072% of unselected cases of prenatal diagnoses, and their molecular cytogenetic characterization is required to establish a reliable karyotype-phenotype correlation. A small group of sSMC are C-band-negative and devoid of alpha-satellite DNA. We report the molecular cytogenetic characterization of a *de novo* analphoid sSMC derived from 18q22.1→qter in cultured amniocytes.

**Results:**

We identified an analphoid sSMC in cultured amniocytes during a prenatal diagnosis performed because of advanced maternal age. GTG-banding revealed an sSMC in all metaphases. FISH experiments with a probe specific for the chromosome 18 centromere, and C-banding revealed neither alphoid sequences nor C-banding-positive satellite DNA thereby suggesting the presence of a neocentromere. To characterize the marker in greater detail, we carried out additional FISH experiments with a set of appropriate BAC clones. The pattern of the FISH signals indicated a symmetrical organization of the marker, the breakpoint likely representing the centromere of an inverted duplicated chromosome that results in tetrasomy of 18q22.1→qter. The karyotype after molecular cytogenetic investigations was interpreted as follows:

47,XY,+inv dup(18)(qter→q22.1::q22.1→neo→qter)

**Conclusion:**

Our case is the first report, in the prenatal diagnosis setting, of a *de novo* analphoid marker chromosome originating from the long arm of chromosome 18, and the second report of a neocentromere formation at 18q22.1.

## Background

Small supernumerary marker chromosomes (sSMC) are structurally abnormal chromosomes that cannot be identified by conventional banding pattern analysis [1]. They are usually characterized by molecular cytogenetic techniques [2]. *De novo* sSMC are not rarely encountered during prenatal diagnosis. In fact, they occur in 0.072% of unselected cases of prenatal diagnoses [3]. Their frequency increases with advanced maternal age [4].

Most *de novo* sSMC originate from acrocentric chromosomes, and approximately 50% of them originate from the centromeric region of chromosome 15 [5]. Karyotype-phenotype correlations are well established in the latter cases [6]. The karyotype-phenotype correlation of the remaining sSMC is generally unknown, and the phenotypes range from normal to dysmorphic features and/or developmental delay, depending on the chromosomal region involved, the level of mosaicism, and tissue distribution of the sSMC [7]. Thus, it is important to characterize new sSMC identified during prenatal diagnosis in order to establish the clinical outcome.

A small group of markers have an analphoid, C-band-negative centromere but they are substantially stable *in vivo* and *in vitro*, which suggests the formation of a newly derived functional centromere, called “neocentromere” [8,9]. We report the finding of a *de novo* analphoid sSMC derived from the long arm of chromosome 18 in a case of prenatal diagnosis, and its characterization using fluorescence *in situ* hybridization (FISH) studies. To our knowledge, this is the second report of the formation of a neocentromere at 18q22.1 [10].

## Case presentation

**Figure 1:**
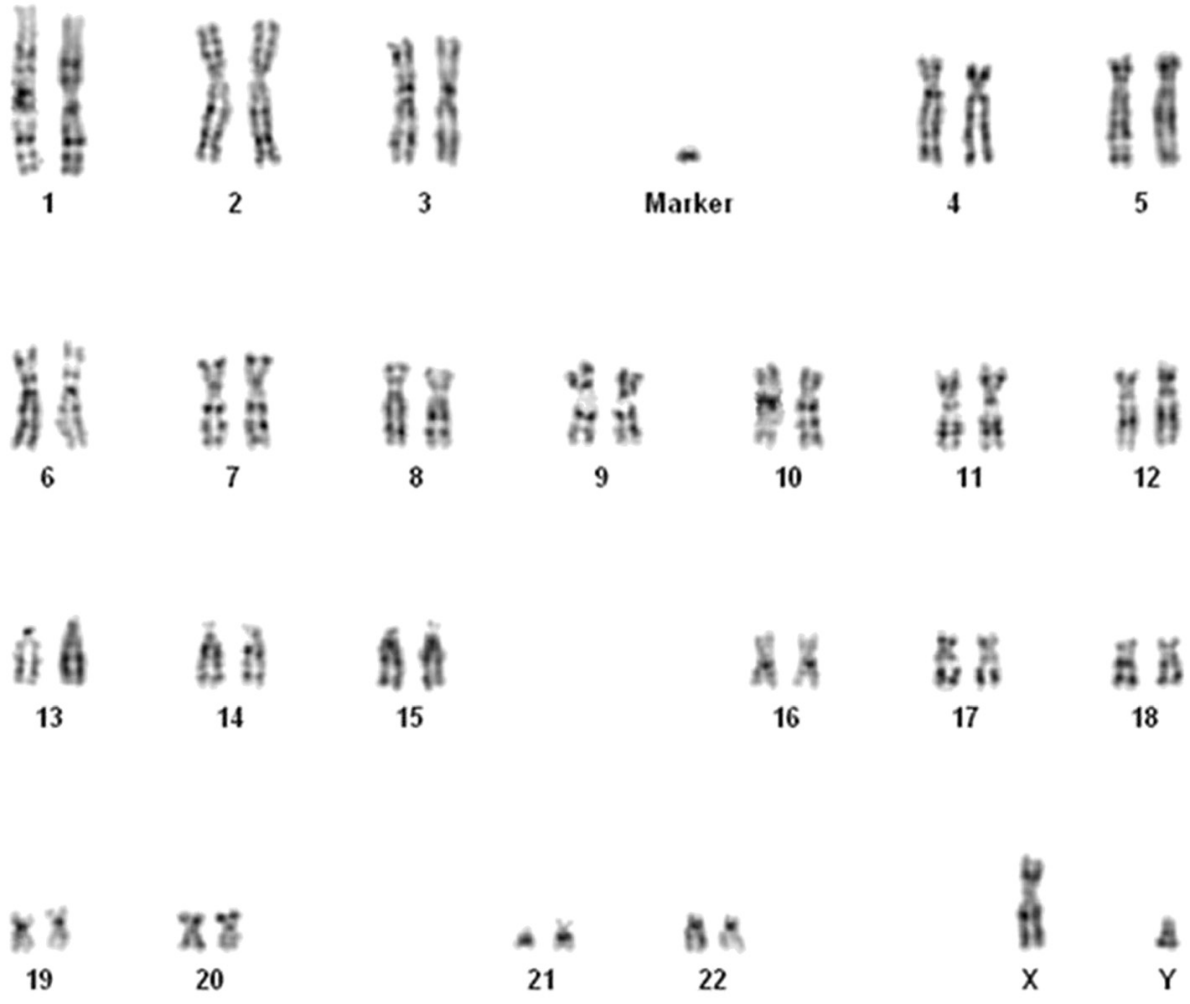
**G-banded karyotype showing the marker chromosome.**

**Figure 2:**
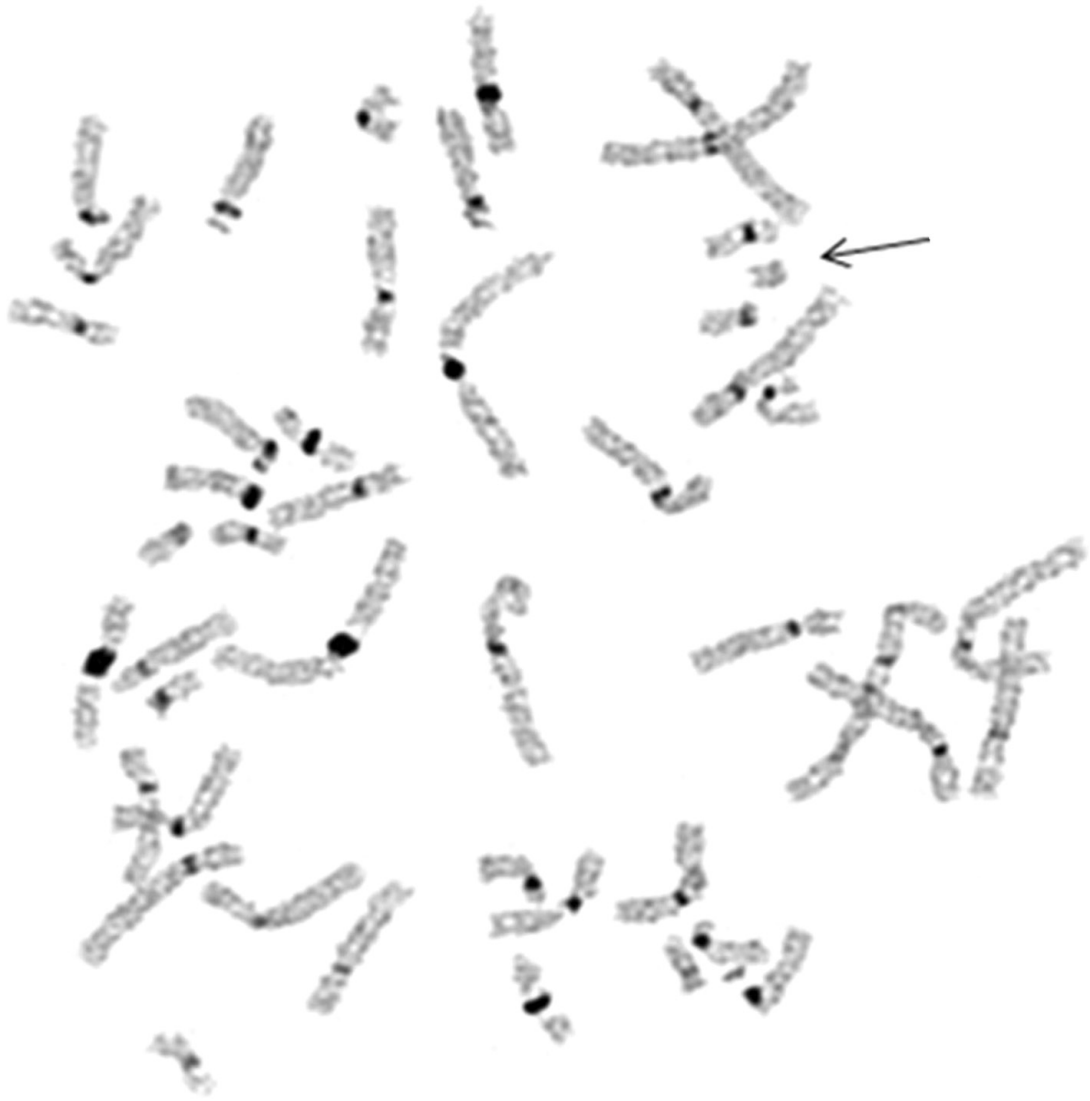
**C-banded metaphase showing the marker chromosome (arrow) with a C-band-negative constriction.**

A 44-year-old primigravida woman underwent genetic counseling and amniocentesis at 18 weeks of gestation because of advanced maternal age. The woman and her husband were healthy and non-consanguineous. There was no family history of congenital malformations. She denied exposure to alcohol, tobacco smoke, irradiation or infectious diseases during pregnancy. The amniotic fluid alpha-fetoprotein level was normal. Rapid screening for aneuploidy in uncultured amniocytes using the BACs-on-Beads™ assay (PerkinElmer Wallac OY, Turku, Finland) did not reveal chromosome anomalies, and fetal gender was male. G-banding cytogenetic analysis, performed on 15 colonies in primary amniocyte cultures, revealed an sSMC about the same size as a group G chromosome in all cells. The fetal karyotype was: 47,XY,+mar (Figure 1). The marker was negative for C-banding (Figure 2). The origin and architecture of the sSMC were unclear.

Chromosome analysis on the two phenotypically normal parents showed normal karyotypes in all the 30 cells examined. Ultrasonography carried out during the 21st week of pregnancy revealed normal fetal growth (30th centile), and nasal bone hypoplasia, which is a soft marker of fetal aneuploidy. The parents were counseled and chose to terminate the pregnancy. A post-mortem examination of the fetus revealed no external dysmorphic features.

**Figure 3:**
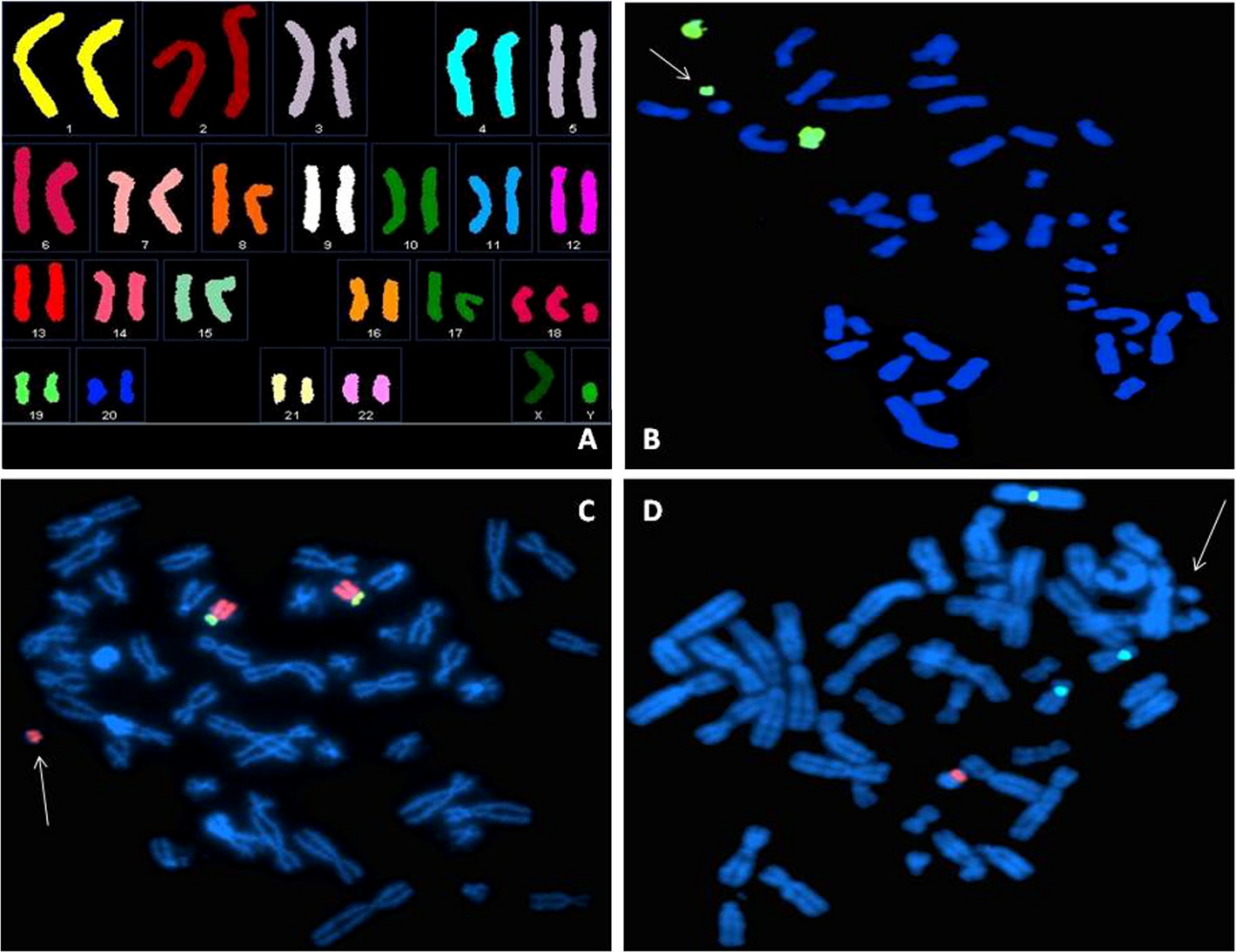
**Molecular cytogenetic characterization of the marker chromosome.** Arrows indicate the marker chromosome. **(A)** Spectral karyotyping. **(B)** Whole chromosome paint 18 green. **(C)** chromosome 18 arm-specific painting, 18p green/18q red. **(D)** Multicolor DNA Probe Kit CEP 18 aqua/X green/Y orange.

Amniocytes were subcultured for further analyses. The marker dropped rapidly to 50%. Multiplex FISH with spectral karyotyping identified the marker as a derivative of chromosome 18 (Figure 3A). This was confirmed using a whole chromosome painting probe specific for chromosome 18 (Figure 3B). Partial chromosome painting probes specific for the long and the short arms of chromosome 18 indicated that the marker derived from the long arm of chromosome 18 (Figure 3C). The multicolor probe panel used to reveal alpha satellite sequences failed to show signals on the marker (Figure 3D). Additionally, we amplified total human DNA with alpha-satellite primers derived from the conserved regions of human alpha satellite sequences [11], and used the amplified product as a probe in FISH experiments. The procedure is able to detect all human centromeres, but it failed to detect any signal on the marker [11]. We therefore assumed that the marker chromosome contained a newly formed centromere (i.e., a neocentromere).

**Figure 4:**
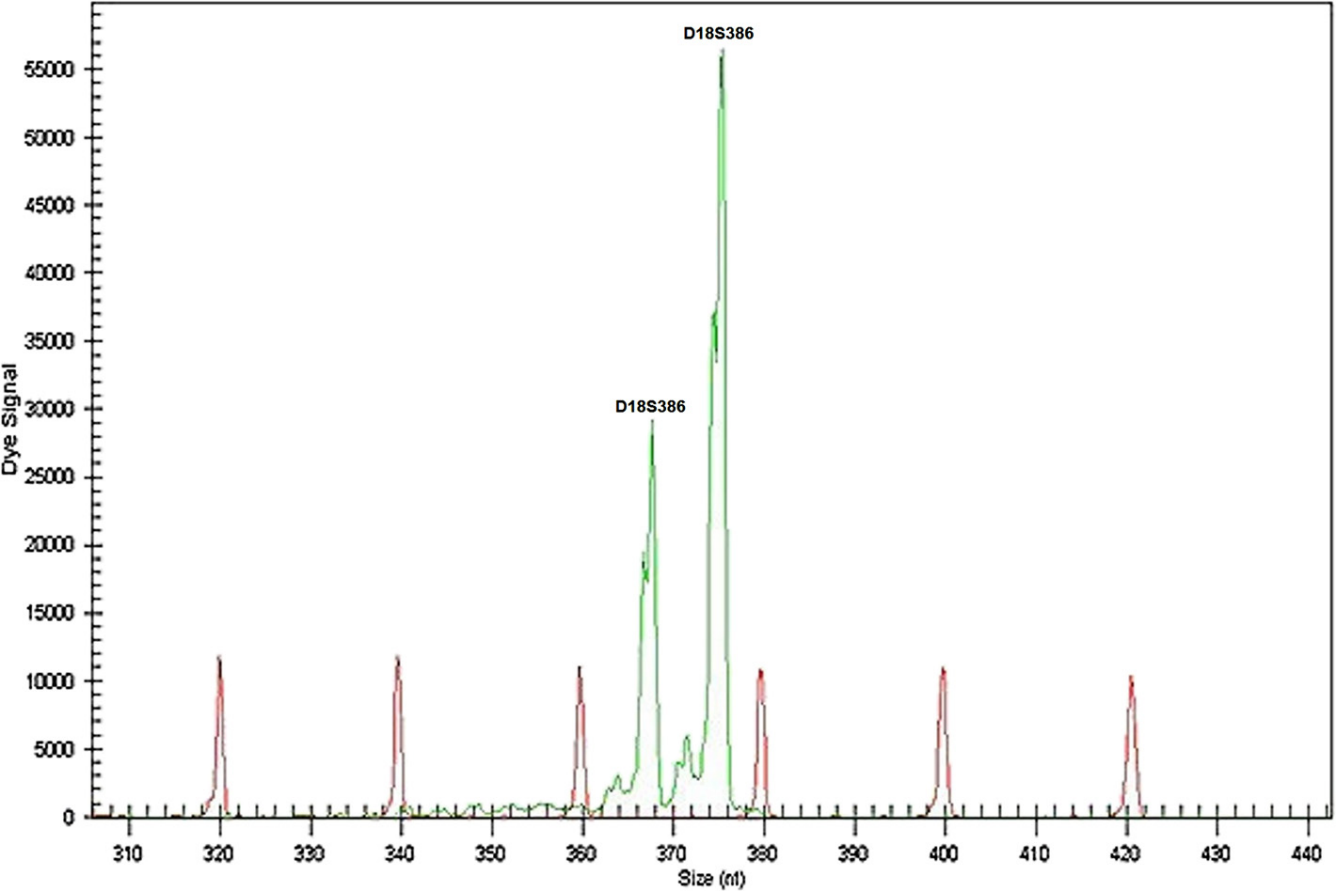
**Electropherogram of the unbalanced marker D18S386 on chromosome 18.**

We performed a quantitative fluorescence-PCR analysis on secondary cultured amniocytes by amplifying 24 loci on chromosomes X, Y, 13, 18 and 21. The electropherograms revealed a male genotype with a normal pattern for chromosomes 13 and 21. In the case of chromosome 18, the informative microsatellite markers D18S391, D18S1002, D18S535, and D18S858 (18pter→18q21.31) showed two balanced peaks, revealing a normal heterozygote pattern, while the D18S386 marker (18q22.1) showed two unbalanced peaks (peak area ratio 1:2) (Figure 4), suggesting that the markers derived from the distal end of the long arm of chromosome 18.

**Figure 5:**
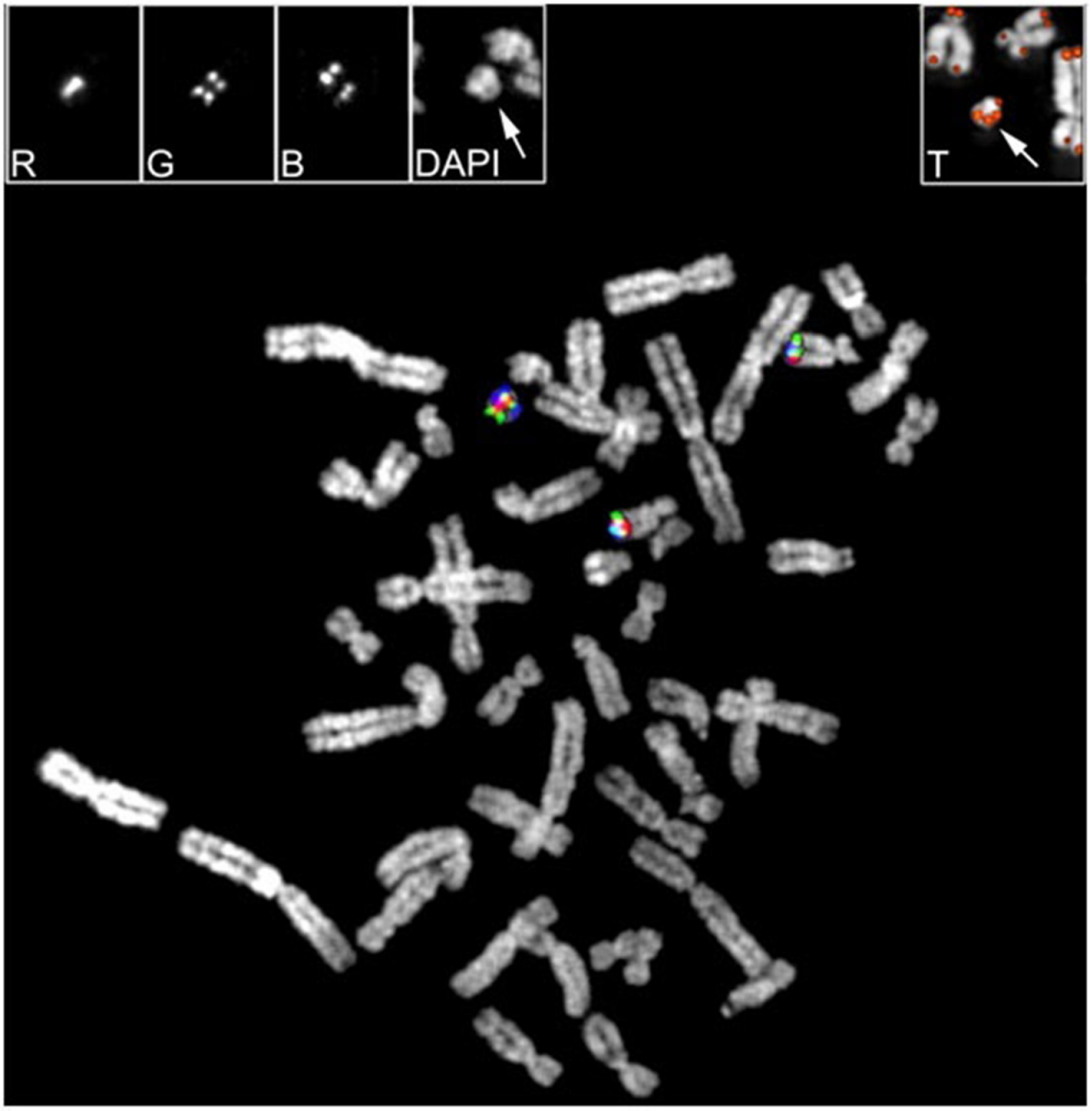
**Co-hybridization experiments using BACs RP11-246I7 (red; centromeric), RP11-177C10 (green) and RP11-715C4 (blue; telomeric).** Their mapping position on chromosome 18 is reported in Table 1. For clarity, the original black and white FISH signals for RP11-246I7 (R), RP11-177C10 (G), RP11-715C4 (B), and DAPI are reported separately in the upper left boxes. The partial metaphase in the upper right box shows the telomeric FISH signals of the marker (arrowed).

We used appropriate BAC clones spanning the 18q21.1→18qter chromosomal region to characterize the extension and organization of the marker in greater detail. Examples of FISH experiments are shown in Figure 5. The exact position of each BAC on the GRCh37/hg19 assembly was identified using the UCSC genome browser (Table 1). The last negative and the first positive BAC clones, RP11-316J22 and RP11-775G23, respectively, defined the breakpoint as lying on, or very close to, the chr18:63,292,882–63,296,150 interval (3,268 bp), at 18q22.1. A FISH experiment with a probe specific for the telomeric sequences revealed a clear signal at both ends of the marker (Figure 5T). The pattern of the FISH signals (Figure 5) indicated a symmetrical organization of the marker, the breakpoint likely representing the centromere of an inverted duplicated chromosome. The latter notion is supported by the finding that CENP-A, playing a key role in centromere specification, is rapidly recruited to double-strand breaks [12].

**Table 1:** **Name, location and FISH results of the BAC clones used in this study**

**BAC name**	**hg19 position**	**Chr band**	**On marker**
RP11-634P8	chr18:47,976,335-48,168,694	18q21.1 - 18q21.2	No
RP11-153B11	chr18:54,667,206-54,826,907	18q21.31	No
RP11-299P2	chr18:60,776,519-60,923,180	18q21.33	No
RP11-933E2	chr18:61,040,533-61,226,049	18q21.33	No
RP11-316J22	chr18:63,126,423-63,292,882	18q22.1	No
Breakpoint			
RP11-775G23	chr18:63,296,150-63,472,757	18q22.1	Yes
RP11-164O16	chr18:63,505,969-63,674,351	18q22.1	Yes
RP11-1069K2	chr18:63,677,806-63,866,636	18q22.1	Yes
RP11-246I7	chr18:64,155,311-64,339,348	18q22.1	Yes
RP11-177C10	chr18:69,512,241-69,673,238	18q22.3	Yes
RP11-715C4	chr18:76,390,026-76,578,392	18q23	Yes

Based on the FISH results we interpreted the karyotype as:

- 47,XY,+inv dup(18)(qter→q22.1::q22.1→neo→qter)

## Conclusions

Supernumerary marker chromosomes are a major clinical problem in prenatal genetic diagnosis and counseling. Detailed characterization of the marker chromosome is required to establish a reliable genotype-phenotype correlation, in the absence of which prognostic counseling can only be based on theoretical data. We report the prenatal molecular cytogenetic characterization of an analphoid sSMC consisting of an inverted duplication of the distal region of chromosome 18q that results in tetrasomy of 18q22.1→qter. In the only other previous report of an apparently neocentric marker of chromosome 18, the authors describe a stable analphoid marker of chromosome 18 in a child with dysmorphic features and mild mental retardation [13]. However, the marker was not characterized in detail.

The severity of the phenotype in sSMC cases could depend on the chromosomal region involved in the aneuploidy, the number of copies of the region, and the stability of the acentric fragment [14,15]. In our case, the couple decided to terminate the pregnancy. The fetus was examined and did not have any apparent anatomical anomalies. However, the high stability of the neocentric sSMC, present in all cultured primary amniocytes, and the euchromatic content suggested a high risk of severe psychomotor retardation. Furthermore, correlative analysis suggested that duplication of 18q22.1→18qter is associated with severe mental retardation [16,17].

Analphoid sSMC are rare but interesting because their survival and stability depend on the seeding of a new centromere known as “neocentromere” [8,9]. Since the pivotal report of Voullaire et al. [18], approximately 120 human neocentromeres, deriving from 20 different autosomal chromosomes as well as from X and Y sex chromosomes, have been described [19]. Most of these neocentromeres are located on marker chromosomes derived from inverted duplications of terminal chromosomal regions, and result in trisomy/tetrasomy of the region [9,20]. The known human neocentromeres cluster in some chromosomal regions [9]. In one case (15q), the cluster region corresponds to the region where an ancestral centromere was inactivated during evolution [21]. In our case, the neocentromere seeding region corresponds, at cytogenetic level, to the same chromosomal 18q22.1 band in which a neocentromere was previously described [10]. In the latter prenatal diagnosis, the centromere repositioned along chromosome 18 that was otherwise normal. It would be interesting to investigate whether or not the same region, at sequence level, underlies both neocentromeres. However, the lack of sequence data in the two cases preclude further investigation. Interestingly, three neocentromeres seeded at 13q32 were found to be seeded in different DNA domains [22].

## Materials and methods

### Chromosome analysis

Chromosome analysis was carried out on metaphase cells derived from amniotic fluid cells according to standard procedures. Twenty metaphase cells from 15 colonies in primary cultures were analyzed with G-banding and C-banding. Chromosome preparations of peripheral blood lymphocytes from the parents were subjected to G-banding, and 30 metaphase spreads were analyzed. Karyotypes are described according to the International System for Human Cytogenetic Nomenclature (ISCN 2013).

### Quantitative fluorescent polymerase chain reaction

DNA from secondary cultured amniocytes was extracted by means of a conventional salting-out protocol. Quantitative fluorescent-PCR was performed with a home-made kit that analyzed, in two multiplex reactions, the amelogenin and SRY genes, and 22 microsatellite markers of chromosomes X, Y, 13, 18 and 21. Capillary electrophoresis was performed with a CEQ 8000 sequencer (Beckman Coulter).

### Fluorescence *in situ* hybridization analysis

We used diverse FISH approaches, namely, multiplex FISH with the Spectral Karyotyping Assay™ (Applied Spectral Imaging Ltd., Migdal Ha’Emek, Israel); a whole chromosome painting probe specific for chromosome 18 (WCP18- Applied Spectral Imaging Ltd., Migdal Ha’Emek, Israel); a partial chromosome paint specific for the long and the short arms of chromosome 18 (MetaSystems, Altlussheim, Germany); a mixture of probes for the X chromosome centromere (DXZ1) and the Y chromosome centromere (DYZ3) (AneuVysion CEP 18/X/Y-alpha satellite; Abbott Molecular Inc., Des Plaines, IL,USA), and a telomere peptide nucleic acids probe (Telomere PNA FISH kit/Cy3; Dako, Denmark). Each FISH procedure was performed according to the manufacturer’s instructions. DNA from BAC clones was extracted, labeled, and hybridized according to Lichter et al. [23] with minor modifications.

## Ethical approval and consent

These studies were performed on anonymized samples received in the clinical laboratory and thus were exempt from the requirement for consent according to the Western Institutional Review Board. However, written informed consent was obtained from the parents for publication of this case report. A copy of the written consent is available for review by the Editor-in-Chief of this journal.
